# The effects of hypoxia on the stemness properties of human dental pulp stem cells (DPSCs)

**DOI:** 10.1038/srep35476

**Published:** 2016-10-14

**Authors:** Nermeen El-Moataz Bellah Ahmed, Masashi Murakami, Satoru Kaneko, Misako Nakashima

**Affiliations:** 1 Department of Stem Cell Biology and Regenerative Medicine, National Center for Geriatrics and Gerontology, Research Institute, Obu, Aichi, Japan; 2 Department of Oro-dental genetics, Division of Human Genetics and Human Genome, National research center, Cairo, Egypt; 3 Reproduction Center, Gynecology, Ichikawa General Hospital, Tokyo Dental College, Sugano, Ichikawa, Chiba, Japan

## Abstract

Recent studies have demonstrated that culture under hypoxia has beneficial effects on mesenchymal stem cells (MSCs). However, there are limitations to achieving a stable condition in conventional hypoxic CO_2_ incubators. DPSCs are a unique type of MSCs which are promising in many regenerative therapies. In this study, we investigated the ideal hypoxic culture environment for DPSCs using a new system that can provide controlled O_2_ environment. The effects of hypoxia (3%, 5%) on the stemness properties of DPSCs. Their morphology, proliferation rate, expression of stem cell markers, migration ability, mRNA expression of angiogenic/neurotrophic factors and immunomodulatory genes were evaluated and compared. Additionally, the effect of the discrete secretome on proliferation, migration, and neurogenic induction was assessed. Hypoxic DPSCs were found to be smaller in size and exhibited larger nuclei. 5% O_2_ significantly increased the proliferation rate, migration ability, expression of stem cell markers (CXCR4 and G-CSFR), and expression of SOX2, VEGF, NGF, and BDNF genes of DPSCs. Moreover, secretome collected from 5%O_2_ cultures displayed higher stimulatory effects on proliferation and migration of NIH3T3 cells and on neuronal differentiation of SH-SY5Y cells. These results demonstrate that 5%O_2_ may be ideal for enhancing DPSCs growth, stem cell properties, and secretome trophic effect.

Mesenchymal stem cells (MSCs) have been evaluated as a potential tool to treat numerous diseases, including tissue injury, degenerative diseases, and immune disorders. This is due to their multipotent differentiation capacity^1^,^2^ trophic activity^3^,^4^, immunomodulatory properties^5^,^6^,^7^ and angiogenic/neurogenic properties^8^. Moreover, MSCs can be efficiently isolated from a wide range of tissues such as bone marrow, adipose tissue, umbilical cord, and dental pulp^9^,^10^,^11^. For research studies and clinical applications, expansion of MSCs *in vitro* is needed in order to obtain sufficient cell numbers. However, poor growth kinetics, early senescence, DNA damage during expansion, poor engraftment, and short-term survival after transplantation are of the major concerns of MSC-based regenerative therapy^12^. Isolation techniques, culture medium, supplements, cell seeding density, oxygen tension, and three-dimensional expansion have been found to possess prominent effects on MSC therapeutic value^13^,^14^. Therefore, it is critical to optimize and standardize the culture conditions of MSCs so that their utility can be recognized in clinical applications.

Oxygen concentration is a critical environmental factor that affects MSCs. It plays an essential role in maintaining stem cell plasticity and proliferation^15^. MSCs are normally cultured *in vitro* in the presence of 5% CO_2_ and oxygen levels of approximately 20%. Natural cell microenvironments, however, contain much lower oxygen tensions ranging from 12% in arterial blood down to 1–7% in a variety of other tissues^16^. In recent years, several studies have presented evidence regarding the negative influence of ambient O_2_ concentration on MSCs, including early senescence^17^, longer population doubling time and DNA damage^18^. On the other hand, 3% O_2_ tension in cell culture had positive effects on the *in vitro* survival and self-renewal of bone marrow stem cells (BMSCs)^19^. While 2% O_2_ tension was found to preserve the stemness and enhance proliferation^20^ and angiogenic potential of adipose-derived MSCs (ADMSCs)^21^. BMSCs were also able to maintain their undifferentiated state when cultured in 3% hypoxia^22^. Moreover, researchers have found that hypoxia is also a critical microenvironmental factor in regulating cancer stem cells. Many studies showed that hypoxia promotes tumor progression, and induce the “dedifferentiation” of differentiated cancer cells which then acquire the stemness^23^,^24^,^25^,^26^

Not only cells cultured under hypoxic conditions show superior properties to those cultured under normoxic ones, but also the secretome collected from hypoxic cultures shows beneficial effects. It has been shown recently that secretome collected from ADMSC cultured under less than 5% O_2_ contains high levels of granulocyte-macrophage-colony-stimulating factor (GM-CSF), vascular endothelial growth factor (VEGF), Interleukin-6 (IL-6), and insulin-like growth factor 1 (IGF-1)^27^ and was also found to be able to protect myocardial infarct in rat^28^.

Dental pulp stem cells (DPSCs) are a unique type of MSCs. Besides their neural crest origin, DPSCs express pluripotent stem cell markers such as; Oct4, Nanog, Sox2, and Klf4 29. DPSCs have more potent neurogenicity and more immunosuppressive activities than other MSCs^30^ Moreover, isolating stem cells from dental pulp is a noninvasive procedure in which the pulp can be collected from either young discarded teeth or from adult wisdom teeth after common surgical extraction procedure. DPSCs were found to be promising in many regenerative therapies such as; dental pulp regeneration^31^,^32^, bone tissue engineering^33^, neurology^34^,^35^, angiogenesis/vasculogenesis^36^,^37^, endocrinology^38^ and healing^39^. Thus, DPSCs can be perfect candidates for cell therapy and future regenerative medicine. Hence, it is preferable to find the optimal culture conditions for DPSCs. Dental pulp tissue is surrounded by hard dentin tissue, and O_2_ reaches the pulpal cells only through the vasculature in root canals. Consequently, the O_2_ tension in dental pulp tissues is lower compared to that in the air^40^. Thus, it is plausible that the ambient O_2_ tension might not be suitable for the establishment and maintenance of DPSCs. In that context, some studies demonstrated that cultivation of DPSCs under hypoxic condition (1–3%) enhances proliferation potential^41^ and angiogenic potential^42^, and affects the odontoblastic differentiation potential^43^.

In general, culturing cells under hypoxia requires a reliable experimental device to maintain a stable hypoxic environment for cell culture. It is difficult to produce very low O_2_ levels in most of the available O_2_/CO_2_ incubators. There are several existing models for such a purpose. One is the modular incubator chamber^44^ that can be filled with the desired gas mixture (O_2_, CO_2,_ and N_2_) prior to placing in the normal culture incubator. That modular incubator chamber is widely used in many research laboratories. One of the common defects of this chamber, however, is leakage which disrupts the experimental processes and sometimes results in uncertainty about actual inner chamber O_2_ concentration. Moreover, the chamber creates inner pressure if the operation is not attained correctly. Another hypoxia model is a cell culture incubator in which O_2_ is displaced by infusing N_2_, which is supplied by an external high-pressure liquid nitrogen tank^45^. Still, each time the incubator door is opened, air flows in and hypoxic conditions are temporarily disturbed and have to be re-established. A third hypoxia model is the tri-gas incubator where a tri-gas mix can be achieved by using either a pre-mixed gas supplied by the provider or by using CO_2_ and N_2_ gases^46^. Tri-gas incubators have a divided or segmented inner chamber with individual glass doors to minimize disturbance of culturing conditions with reduced operating costs that can help conserve gasses and reduce the chances of contamination. Unfortunately, all the available hypoxia models can only provide one hypoxic level at a time. They are also quite expensive, and they may be inconvenient for small laboratories that do not do hypoxia experiments often. That is why, in most of the previous studies, MSCs were cultured under a single hypoxic condition and they could not compare more than one condition at the same time. In addition, only a few studies have reported the effect of continuous O_2_ tension in long-term culture of MSCs starting from the primary culture^19^,^47^. Moreover, the incubators used have not been designed for strict control of O_2_ throughout the culture.

Therefore, in the present study, we have modified the conventional tri-gas incubator in order to supply stable O_2_ concentration and manipulate microenvironment continuously during the culture period of DPSCs starting isolation^48^. Then, we examined the effect of different oxygen concentrations (20%, 5%, and 3%) on DPSC biology including; stem cell phenotype, proliferation and migration ability, expression of trophic factors and trophic effects. We provide evidence that 5% O_2_ hypoxia may be optimal for expanding DPSCs.

## Results

### Hypoxic cultures exhibit better stem cell morphology

**Figure 1:**
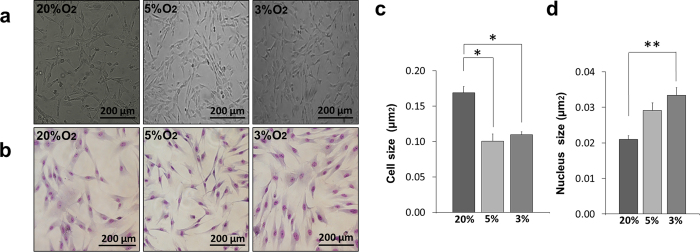
DPSCs exhibited better morphology under hypoxic conditions. (**a**) Representative phase contrast micrographs of DPSCs (5^th^) cultured in different oxygen tension. (**b**) Representative micrographs of DPSCs (5^th^) cultured in different oxygen tensions stained with Giemsa stain. (**c**) Cell size as analyzed by Image J. (**d**) Nucleus size as analyzed by Image J. (mean ± S.E., n = 4. *p < 0.05, ***p < 0.001).

Primary cells were successfully isolated from the dental pulp of four donors. Isolated DPSCs were able to attach to culture plates and expand *in vitro* in all normoxic (20% O_2_) and hypoxic (5%, and 3% O_2_) cultures. At early passages, cells under all three oxygen conditions exhibited small, spindle-shaped morphologies. The morphology of the isolated cells, however, became considerably different between normoxia and hypoxia in further passages. DPSCs under normoxia showed a larger and more flattened morphology than those under hypoxia (Fig. 1a). Also, Giemsa staining showed that hypoxic cells exhibited larger nuclei than normoxic cells (Fig. 1b). Upon trypsinization, it was harder to detach DPSCs in hypoxic cultures and took a longer time to obtain single cell suspensions from the detached cells compared to DPSCs in normoxic cultures.

Further analysis of cell morphology images demonstrated that DPSCs cultured in normoxic condition are statistically larger in size (0.2 μm^2^ ± 0.01 μm^2^, p < 0.05, mean ± S.E) than DPSCs cultured in hypoxic conditions; 5% (0.1 μm^2^ ± 0.01 μm^2^) and 3% O_2_ (0.1 μm^2^ ± 0.003 μm^2^) (Fig. 1c). Moreover, Giemsa staining revealed that DPSCs cultured under 3% O_2_ had statistically larger nuclei (0.03 μm^2^ ± 0.002 μm^2^, p < 0.01) compared to DPSCs cultured in 20% (0.02 μm^2^ ± 0.001 μm^2^). While DPSCs cultured in 5% O_2_ (0.03 μm^2^ ± 0.002 μm^2^) showed no significant differences in nucleus size compared to DPSCs cultured in 20% and 3% oxygen (Fig. 1d).

### Expression of stem cell markers is higher in hypoxic cultures

**Figure 2:**
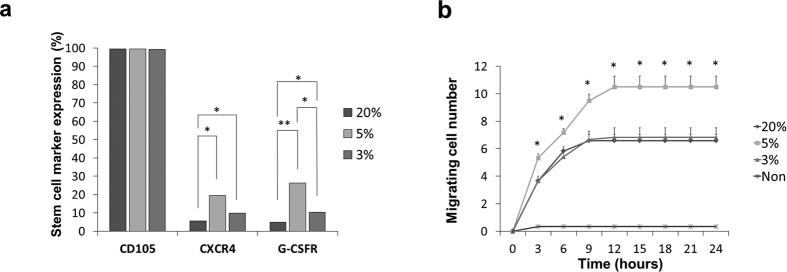
5% oxygen cultures exhibited higher stem cell surface markers expression and migration ability. (**a**) Expression of surface stem cell markers. (**b**) Migrating cell number. (mean ± S.E., n = 4. *p < 0.05, **p < 0.01).

All normoxic and hypoxic cultures at 5th passage expressed mesenchymal stem cell marker CD105, equally approaching 100% when analyzed by flow cytometry. While, the expression of another stem cell marker; CXCR4 was significantly higher in 5% O_2_ (19.55% ± 0.03) and 3% O_2_ (9.78% ± 0.01) cultures than in 20% O_2_ cultures (5.68% ± 0.01, p < 0.05). There was no significant difference in CXCR4 expression between 5% O_2_ and 3% O_2_ cultures. On the other hand, G-CSFR expression was significantly higher at 5% O_2_ (26.23% ± 0.02) than at 20% O_2_ (5.00 ± 0.01, p < 0.01) and at 3% O_2_ (10.20% ± 0.01, p < 0.05). Likewise, there was a significant difference in G-CSFR expression between 3% cultures and 20% cultures (p < 0.05) (Fig. 2a). Numerical analysis for the percentages of cells expressing stem cell markers can be found as Supplementary Table S1.

### Migration capability is higher in 5% O_2_ cultures

The migration ability towards G-CSF was examined in normoxic and hypoxic cultures at the 5th passage. DPSCs from hypoxic cultures especially at 5% O_2_ were able to migrate at a higher rate compared to DPSCs from normoxic cultures. There was a significant difference between the number of migrating cells from 5% O_2_ cultures and those migrating from 3% O_2_ cultures at; 3, 6 hours, 9 hours and at 12 hours (p < 0.05). On the other hand, there was no significant difference between the migrating cell number from 3% O_2_ and 20% O_2_ cultures at any time interval (Fig. 2b). No more cells were able to migrate after 12 hours.

### Cell proliferation is enhanced in 5% O_2_ cultures

**Figure 3:**
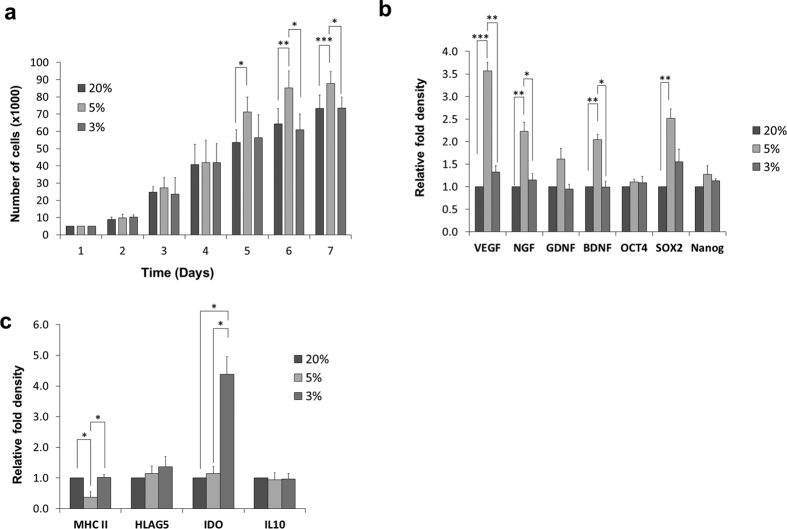
Hypoxic cultures demonstrated better proliferation rate, higher expression of pluripotency markers, angiogenic/neurotrophic factors, and immunomodulatory genes. (**a**) Cell count. (**b**) Quantitative RT-PCR for mRNA levels of pluripotency markers and angiogenic/neurotrophic factors. (**c**) Quantitative RT-PCR for mRNA levels of immunomodulatory genes (mean ± S.E., n = 4. *p < 0.05, **p < 0.01, ***p < 0.001).

Proliferation rate was evaluated at the 3rd culture passage. We found that the rate of cell growth was same for all normoxic and hypoxic cultures at the start of culture. However, 5% O_2_ cultures demonstrated higher cell numbers than 3% O_2_ and 20% O_2_ cultures starting day 5 (Fig. 3a). That increase in the cell proliferation rate of 5% O_2_ cultures became more significant as the culture time was extended; on day 6 and 7. There was a significant difference in the proliferation rate between 3% O_2_ and 5% O_2_ but not between 3% O_2_ and 20% O_2_.

### Expression of pluripotency markers, trophic factors, and immunomodulatory genes

Hypoxia did not lead to enhanced mRNA levels of pluripotency markers; Nanog and Oct4. However, the relative fold expression of Sox2 was significantly increased in 5% O_2_ cultures (2.5 ± 0.2, p < 0.01) compared to 20% but not significant when compared to 3% O_2_ cultures. Angiogenic trophic factor (VEGF) expression was generally higher in hypoxia than normoxia but was significantly upregulated in 5% O_2_ cultures (3.6 ± 0.2) compared to 20% (p < 0.001) and 3% O_2_ cultures (1.3 ± 0.13, p < 0.01). Moreover, neurotrophic factors (BDNF and NGF) expression was significantly increased in 5% O_2_ cultures (2.04 ± 0.1 and 2.2 ± 0.2, respectively, p < 0.01) compared to 20% O_2_ cultures as well as when compared to 3% O_2_ cultures (1 ± 0.1 and 1 ± 0.1, respectively, p < 0.05). Furthermore, GDNF expression was higher in 5% O_2_ than in the other 2 oxygen conditions but without significance (Fig. 3b). The immunomodulatory factor IL10 was expressed equally in hypoxic and normoxic cultures. However, hypoxia upregulated the mRNA levels of another immune-related factor, IDO with marked significance in 3% O_2_ cultures (4.3 ± 0.6, p < 0.05) compared to 5% and 20% O_2_ cultures. On the other hand, 5% hypoxia significantly decreased gene expression of MHC-II compared to 3% O_2_ and 20% O_2_ cultures (p < 0.05) (Fig. 3c).

### The trophic effect of the secretome is enhanced in 5% O_2_ cultures

**Figure 4:**
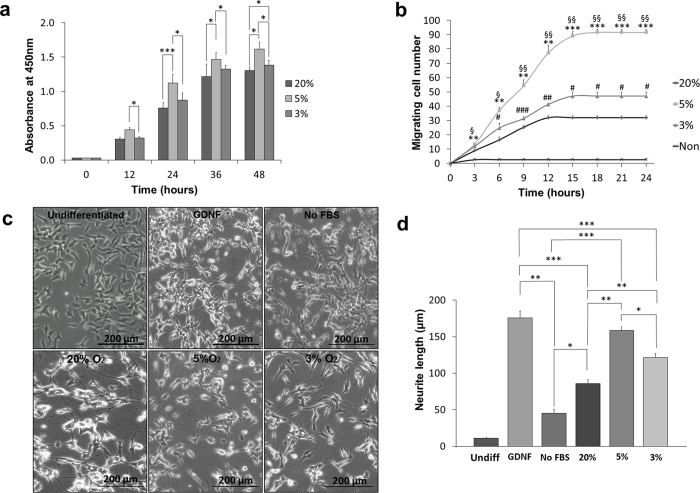
Secretome collected from 5% hypoxic cultures showed better effect on proliferation activity, migration ability, and neurite outgrowth. (**a**) Proliferation activity(*p < 0.05, ***p < 0.001) and (**b**) The migratory activity of NIH3T3 cells supplemented with secretome of each oxygen culture, (mean ± S.E., n = 4) 5% vs. 20% (*p < 0.05, **p < 0.01, ***p < 0.001), 3% vs. 20% (^#^p < 0.05, ^##^p < 0.01, ^###^p < 0.001), 5% vs. 3% (^§^p < 0.05, ^§§^p < 0.01) (**c**) Representative photos demonstrating the morphology of SH-SY5Y supplemented with secretome of each oxygen culture. (**d**) Quantitative analysis of SH-SY5Y neurite outgrowth. (mean ± S.E., n = 4, *p < 0.05, **p < 0.01, ***p < 0.001).

The impact of DPSC secretomes derived from normoxic and hypoxic conditions was evaluated on the proliferation of NIH3T3 cells using cell counting kit-8 (CCK-8). Hypoxia was able to promote NIH3T3 cells proliferation better than normoxia. The increase of proliferation rate was significant between 5% and 20% O_2_ cultures at 24 hours (p < 0.001), 36 hours (<0.05) and at 48 hours (p < 0.05). Moreover, the proliferation rate was significantly higher in 5% O_2_ cultures compared to 3% O_2_ at 12 hours, 24 hours, 36 hours and 48 hours (p < 0.05). On the other hand, there was a significant difference only in the proliferation rates between 3% and 20% O_2_ cultures at 48 hours (p < 0.05) (Fig. 4a).

Secretome of the 5% O_2_ cultures was more effective in enhancing the migration ability of NIH3T3 when compared to 3% and 20% O_2_. There was a significant difference in the number of migrating cells between 5% O_2_ and 3% O_2_ secretomes at 6 hours, 9 hours, 12 hours, and 15 hours. Similarly, the number of migrating cells was significantly higher towards 5% O_2_ secretome compared to 20% O_2_ secretome at 3 hours, 6 hours, 9 hours, 12 hours and 15 hours. Likewise, the number of migrating cells towards 3% O_2_ secretome was significantly higher than that towards 20% O_2_ at 6 hours, 9 hours, 12 hours and 15 hours. No more cells were able to migrate after 15 hours (Fig. 4b).

Neurite outgrowth in human neuroblastoma SH-SY5Y cells was also enhanced by exposure to hypoxic culture secretome compared to normoxic one. Nevertheless, the secretome of 5% O_2_ cultures was significantly more effective on neurite outgrowth than that of 3% O_2_ cultures and 20% O_2_ cultures. It also worth mentioning that, when comparing the neurite outgrowth enhanced by the glial cell line-derived neurotrophic factor (GDNF) and that enhanced by the secretome of 5% O_2_ cultures, there was no significant difference, while GDNF significantly enhanced neurite outgrowth of neuroblastoma cells compared to the secretomes of 3% O_2_ and 20% O_2_ cultures (Fig. 4c,d).

## Discussion

Whereas the effects of hypoxia on DPSCs have been investigated previously, the optimal hypoxic culture conditions and the methods to achieve it remain unknown. The optimization of DPSCs culture conditions is of great importance for expanding DPSCs with high quality for more successful application in regenerative medicine. Thus, in the present study, we introduced a new hypoxia system to supply stable O_2_ tension during the culture period starting from primary culture and demonstrated that 5% O_2_ tension may provide an excellent culture condition for DPSCs. As early as 1958, it was discovered that animal cells proliferate more rapidly in O_2_ concentrations lower than ambient oxygen^49^. Since that time, substantial progress has been made in understanding cell responses to decreased O_2_ concentrations and the mechanisms underlying this response. However, data from various studies are difficult to compare due to wide variations in O_2_ tension, duration of culture, the use of MSC from different species and different media composition^47^,^18^. Moreover, different cell types vary in their capacity to cope with hypoxia over extended periods of culture, and the degree of their adaptive abilities may reflect their native O_2_ microenvironments^50^. Therefore, it is crucial to find the suitable oxygen culture condition for each cell type. Accordingly, we cultured and compared DPSCs in different oxygen tensions (20%, 3%, and 5%) in an attempt to determine which will be physiological for DPSCs culture and expansion.

In order to provide the cells with an ideal hypoxic condition, a well-controlled hypoxia chamber should be established; easy to use, stable and leakage-free to minimize phenotypic changes within the cells. The chamber is expected to create an accurate low O_2_ experimental setting and quick recovery from any disturbances. In the present study, we validated a novel chamber for hypoxia experiments. The chamber provides an accurate experimental hypoxic setting. It provides practical, controlled, and isolated atmospheres for cell cultures. Because of its small size and the fact that it does not require complicated flushing procedures, the newly introduced culture chambers are extremely efficient in gas use as it is not necessary to use the entire incubator for an experiment. Only as many plates as required can be cultured in precise hypoxia conditions with minimal gas usage. Moreover, they reach set point quickly as their recovery time is remarkably fast.

The morphology of MSCs is usually correlated with their “quality”. Many studies have reported that smaller cells have higher self-renewal capacity and better differentiation potential^51^. In that context, other studies showed that mesenchymal stem cells show better morphology and exhibit smaller size when cultured in hypoxia^52^. Agreeing with those studies, our data confirmed that cells cultured in hypoxia exhibited better morphology and smaller size than those cultured in normoxia. We also found that, upon characterization, hypoxia induced the expression of stem cell markers CXCR4 and G-CSFR at a higher rate than normoxia. This coincides with some previous studies^47^,^53^, while contradicts with others which demonstrated that the cell surface phenotype of MSCs was almost unaffected^54^. The CXCR4/SDF-1 axis has been studied by a number of researchers explaining their role in stem cells homing to a specific tissue^55^,^56^. This pathway has been found to be important for regeneration of various damaged tissues^57^. Similarly, the effect of G-CSFR on the mobilization of MSCs has been well established^58^. Moreover, previous studies showed that hypoxia enhanced the migration capacity of MSCs when evaluated by *in vitro* migration assay^59^. In the present study, we demonstrate that 5% hypoxia induces a significant increase in expression of CXCR4 and G-CSFR and thereby controls stem cell trafficking and migration. Hence, the migration ability of 5% O_2_ cultures was significantly higher than that of 3% and 20% O_2_ cultures. This enhanced migration capacity has a clinical implication as these hypoxic cells will be better at homing and targeting a specific tissue which may lead to better engraftment *in vivo*.

When we evaluated the proliferation rate of the cultured DPSCs, we found that DPSCs grew under hypoxia likewise under normoxia on the first 4 days of culture that could be considered as an initial period of adaptation to hypoxic conditions. Then, the proliferation rate of DPSCs was higher in hypoxia on the following days. These results match previously published results^37^,^59^,^60^. However, our results showed clearly that 5% O_2_ significantly enhances proliferation of DPSCs better than 3% and 20% O_2_.

Moreover, in the present study, hypoxia upregulated the gene expression of pluripotency markers; Oct4, Nanog, and Sox2, these results agree with other studies that have described up-regulation of pluripotency markers of hypoxic MSCs^18^,^21^,^61^. Nevertheless, 5% O_2_ increased the expression of pluripotency marker Sox2 significantly more than 3% and 20% O_2_. Additionally, the expression of the angiogenic trophic factor VEGF, as well as the neurotrophic factors BDNF and NGF was significantly increased in 5% O_2_. We further found that hypoxia does not hinder the immunosuppressive properties of DPSCs as the expression of HLA-G and IL-10 genes was not changed under hypoxia. However, cells cultured in 5% hypoxia showed significantly lower expression of MHC-II compared to 3% and 20% O_2_, while cells cultured in 3% hypoxia cultures showed the highest expression of IDO. These results indicate that the immunosuppressive properties of DPSCs are not only maintained at low oxygen concentrations, as previously described in some studies^61^ but are also enhanced. This enhancement of the immunosuppressive properties will provide cells which are even better and more suitable for allogeneic transplantation.

Our data showed that the secretome collected from hypoxic cultures has enhanced the proliferation and migration of NIH3T3 cells, as well as the neurite outgrowth of SH-SY5Y cells. These results are in line with what was previously reported regarding the effect of hypoxia on the trophic effects of collected secretomes^62^,^63^. However, our data demonstrates that the secretome collected from 5% O_2_ had the preeminent effect when compared to 3% and 20% O_2,_ indicating that it may be the best hypoxic culture condition for DPSCs.

In conclusion, since, oxygen tension is a critical parameter, possibly the most important one, in the culture of stem cells, fine-tuning of O_2_ tension to the specific stages of *in vitro* culture may produce cells superior in their characters. Here, we introduced new equipment that can provide stable hypoxic culture condition and demonstrated that 5% O_2_ tension can be considered an excellent culture condition for DPSCs. 5% O_2_ DPSC cultures exhibit better morphology, express stem cells markers at higher rates, have stronger migration ability and better proliferation rate than 3% and 20% O_2_ cultures. Moreover, 5% O_2_ increased the expression of some pluripotency markers, trophic factors, immunomodulatory genes and enhanced the trophic effect of the collected secretome. The above information provides a promising solution to obtain high-quality DPSCs for the needs of regenerative therapy.

## Methods

### Isolation of dental pulp stem cells (DPSCs)

Normal human third molar teeth indicated for extraction were collected from patients aged 20–28 years at the Aichi-Gakuin University Dental Hospital, in accordance with the approved guidelines set by the School of Dentistry, Aichi-Gakuin University, and the National Center for Geriatrics and Gerontology Research Institute. All experimental protocols were approved by the National Center for Geriatrics and Gerontology Research Institute. Informed consent was obtained from all subjects involved in this experiment and donor information for used DPSCs can be found as Supplementary Table S2. The pulp was gently removed using a sterile dental probe and the collected pulp tissue was dissected and digested in 0.2% Liberase MNP-S enzyme (Roche, Germany). The isolated dental pulp cells (DPCs) were cultured in Dulbecco modified eagle’s medium (DMEM) supplemented with 10% human serum collected from healthy consenting adult donors and Antibiotic-Antimitotic solution (life technologies) containing; 100 units/mL of penicillin, 100 μg/mL of streptomycin, and 0.25 μg/mL of Fungizone®, final concentration. Cells were selected on the basis of their ability to adhere to the dish; non-adherent cells were removed during medium replacement after 4–5 days in culture. DPSCs from up to 4 donors were used to conduct the various assays.

### Culture of DPSCs under various oxygen tensions

**Figure 5:**
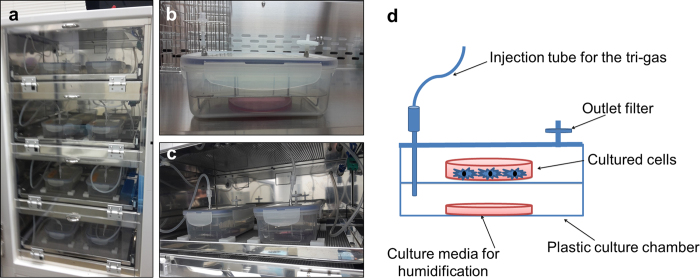
Modified tri-gas incubator for stable hypoxic cultures. (**a**) Tri-gas incubator with 4 segmented chambers. (**b**) Culture box with outlet filter and connector to tri-gas mixture injection tube. (**c**) Assembled culture boxes inside the tri-gas incubator. (**d**) A schematic diagram for the modified culture chamber.

For the normoxic cultures, DPSCs were cultured at 95% air (20% O_2_)–5% CO_2_ in a normal incubator. While, for the hypoxic cultures, DPSCs were cultured in a tri-gas incubator (AIRTECH, Tokyo, Japan). Cells were cultured in dishes and put in particularly designed culture chambers. The novel culture chamber is formed of a plastic box that is connected to an outlet filter and a tube through which premixed gas of O_2_, CO_2_ and N_2_ gasses was continuously injected. Each culture chamber was flushed with humidified gas mixtures of the composition of either (3% O_2_-6% CO_2_-91% N_2_) or (5% O_2_-6% CO_2_-89% N_2_) (Eba Co., Ltd., Nagoya, Japan). A Petri dish; filled with culture media was put in the culture box to provide adequate humidification of the cultures and then the culture box lid was closed. The culture chamber was then returned to the tri-gas incubator and connected to the injection tube (Fig. 5a–c). The PH of the hypoxic cultures was adjusted by adding HEPES buffer (Gibco) at a final concentration of (10 mM).

### Morphological assessment and analysis of cell and nucleus size

Morphological changes were observed under an inverted light microscope (Leica, 6000B-4) using Suite V3 (Leica). Cells were regularly monitored using phase-contrast microscope and images were captured for analysis. Cells at the 3rd passage were prepared for Giemsa staining by; aspirating the old media, rinsing each dish twice with PBS and then fixing the monolayer with absolute methanol for 5 min. The methanol was then poured off and the plate was air-dried. The cells were stained by adding 1 mL of Giemsa reagent (Sigma). Dishes were incubated at room temperature for 20 min and then rinsed extensively with distilled water. The dishes were then observed under an inverted light microscope (Leica, 6000B-4) using Suite V3 (Leica). For cell and nucleus size assessment, multiple representative fields of cells were photographed with an inverted light microscope (Leica, 6000B-4). Captured images were labeled with a scale according to the correspondent microscope magnification (×10). The images scale was used to convert pixels units into micrometers (μm), using Image J software (version 1.49r15). The size of 5 to 10 cells and their nuclei per field was traced and measured.

### Flow cytometric analysis

Cells at the 5th passage of culture in each oxygen tension were detached from flasks using Accutase (Sigma-Aldrich). For identification of stem cell surface markers, cells (1 × 10^5^) were labeled with antibodies against the surface markers CD105 (FITC, mouse, Abcam, Cambridge, UK), CXCR4 (FITC, mouse, R&D systems, USA), G-CSFR (FITC, mouse, R&D systems, USA), IgG1 isotype control (FITC, mouse, Biolegend, USA) and IgG2a isotype control (FITC, mouse, Santa Cruz, USA) for 1 hour, at 4 °C. Labeled cells were analyzed using FACSAria II flow cytometer (Becton Dickinson, USA). Only viable cells as determined by propidium iodide (PI) (Sigma-Aldrich) exclusion were gated and analyzed.

### Cell migration and proliferation assays

At the 5th passage of culture, the migration ability of hypoxic cells towards G-CSF was compared to that of normoxic cells, by a horizontal chemotaxis assay using TAXIScan-FL (Effector cell institute, Tokyo, Japan). The TAXIScan-FL consists of an etched silicon substrate and a flat glass plate, both of which form two compartments with a 6 mm deep micro-channel. Each cell fraction (1 × 10^5^cells/ml) was injected into a single hole, and 1 mL of 10 ng/mL of G-CSF was injected in the contra-hole. Images of cell migration were taken for 24 hours and the number of migrating cells was counted.

For assessment of proliferation activity, the 3rd passage of cells cultured under the three oxygen conditions was allowed to proliferate for 7 days. The samples were harvested in duplicate for 7 consecutive days. Each day of culture, the cells were trypsinized, resuspended, and counted in a hemacytometer.

### Real-time polymerase chain reaction

Total RNA was extracted from normoxic and hypoxic DPSCs at the 5th passage using Trizol kit (Invitrogen, USA). First strand cDNA was generated using 1 μg of total RNA. Primers for the stem cell markers; Oct-4, Sox2, and Nanog and for the angiogenic/neurotrophic factors; vascular endothelial factor (VEGF), nerve growth factor (NGF), brain-derived neurotrophic factor (BDNF), and glial cell-line derived neurotrophic factor (GDNF), as well as primers for immunomodulatory genes; indoleamine2,3-dioxygenase (IDO), interleukin 10 (IL10), Major histocompatibility complex class II (MHC-II) and human leukocyte antigen G5 (HLA-G5) were used to produce PCR products labeled with light cycler-fast start DNA master SYBR Green I (Roche Diagnostics, Switzerland) in a light cycler (Roche Diagnostics).The expression levels in normoxic cells were compared with that of hypoxic cells after normalization to β-actin. The sequences of primers are listed in Supplementary Table 3.

### Assessment of trophic effects of collected secretome

Cells at passages (4th–5th) were grown to 60% confluency, then culture medium was switched to DMEM without serum and cells were starved for 24 hours. The medium from each condition was then collected and concentrated approximately 40-folds by Amicon Ultra-15 centrifugal filter unit with an ultracel-3 membrane (Millipore, Billerica, MA). Halt protease inhibitor cocktail (Thermo Scientific Inc., Waltham, MA, USA) was added to the collected secretome at a concentration of 10 μl/ml. Protein concentration was measured by Coomassie (Bradford) protein assay kit (Thermo Scientific Inc.). The collected secretome was either used immediately or frozen at −30 °C up to one month.

To compare and analyze the enhanced effect of DPSC secretome collected from cells cultured under different oxygen tension on proliferation, mouse embryonic fibroblast cells (NIH3T3) (JCRB, Tokyo, Japan) were cultured in DMEM supplemented with 10% FBS for 24 hours. Then, the medium was changed into DMEM, containing each secretome at a final concentration of 5 μg/ml protein. Cell numbers were measured by cell counting kit 8 (CCK8) (Dojindo Laboratories, Kumamoto, Japan). Cell numbers were measured at 12, 24, 36 and 48 hours. The absorbance at 450 nm was determined by a multiplate reader (Appliskan Multimode) (Thermo Scientific Inc.). Mean values of the mean absorbance rates from four wells were calculated.

To examine the stimulatory effect of each collected secretome on NIH3T3 cell migratory activity, a horizontal chemotaxis assay was performed by using TAXIScan-FL (Effector cell institute, Tokyo, Japan) as previously described. Secretome collected from each oxygen culture was added at 5 μg/ml in the contra-hole. The video images of cell migration were taken for 24 hours.

The enhanced effect of each collected secretome on neurite outgrowth was assessed in human neuroblastoma SH-SY5Y cell line (DS Pharma Biomedical Co. Ltd., Osaka, Japan). SH-SY5Y cells were cultured in DMEM/ham F12 (sigma) supplemented with 10% FBS and Antibiotic-Antimitotic solution (life technologies) for 24 hours. For quantification of neurite outgrowth, SH-SY5Y cells were serum starved and stimulated with secretome from cells cultured in 20%, 5% and 3% O_2_ (5 μg/ml) or with GDNF (Peproteck, London, UK) (20 ng/ml) for 48 hours. In order to assess neurite lengths, multiple representative fields of cells morphology were photographed with an inverted light microscope (Leica, 6000B-4). Captured images were labeled with a scale according to the correspondent microscope magnification (×10). The images scale was used to convert pixels units into micrometers (μm), using Image J software (version 1.49r15). The length of 5 to 10 neurites per field was traced and measured.

### Statistical analysis

Data are reported as mean ± standard error. Statistical analysis was performed using Microsoft (MS) Office Excel Software. One way ANOVA was used to assess for differences between groups and p-values were calculated using unpaired Student’s t-test using IBM SPSS version 19. Differences were considered statistically significant if the p-value was less than 0.05. The number of replicates in each experiment is indicated in the figure legends.

## Additional Information

**How to cite this article**: Ahmed, N. E.-M. B. *et al*. The effects of hypoxia on the stemness properties of human dental pulp stem cells (DPSCs). *Sci. Rep*. **6**, 35476; doi: 10.1038/srep35476 (2016).

## Supplementary Material

Supplementary Information
