# Supplementary material for: The effects of hypoxia on the stemness properties of human dental pulp stem cells (DPSCs)
Source: Sci Rep. 2016 Oct 14;6:35476. doi: 10.1038/srep35476 (PMC5064411; doi:10.1038/srep35476)
Supplement: Supplementary Information [file srep35476-s1.pdf]

Title:

The effects of hypoxia on the stemness properties of human dental pulp stem cells (DPSCs)

Authors:

Nermeen EL-Moataz Bellah Ahmed[1,3], Masashi Murakami[1], Satoru Kaneko[2] & Misako Nakashima[1]

Affiliations

[1]Department of Stem Cells and Regenerative Medicine, Center of Advanced Medicine for Dental and Oral Diseases, National Center for Geriatrics and Gerontology, Research Institute, Obu, Aichi, Japan

[2]Reproduction Center, Gynecology, Ichikawa General Hospital, Tokyo Dental College, Sugano, Ichikawa, Chiba, Japan

[3]Department of Oro-dental genetics, Division of Human Genetics and Human Genome, National research center, Cairo, Egypt

- **Supplementary Table 1. Numerical analysis for the percentages of cells expressing stem cell markers analyzed by flow cytometry.**
- **Supplementary Table 2. Donor information for used dental pulp derived mesenchymal stem cells.**
- **Supplementary Table 3. Human primer sequences used in real-time polymerase chain reaction analysis**

Table S1. Numerical analysis for the number of cells analyzed by flow cytometry and their percentages

	CD105	CXCR4	G-CSFR
**20% O_2**	99.58 %	5.68 %	5.00 %
**5% O_2**	99.48 %	19.55 %	26.23%
**3% O_2**	99.33 %	9.78 %	10.20%

Table S2. Donor information for used dental pulp derived mesenchymal stem cells.

**ID**	#1	#2	#3	#4
**Sex**	Female	Female	Female	Female
**Age**	22	25	23	21
**Tooth**	Upper right third molar	Upper left third molar	Lower left third molar	Lower right third molar

Table S3. Human primer sequences used in real-time polymerase chain reaction analysis

Gene	Primer sequence		NCBI reference
B-actin	5’-GGACTTCGAGCAAGAGATGG-1’<br>3’-AGCACTGTGTTGGCGTACAG-2’	Forward<br>Reverse	NM_001101
BDNF	5’-AAACATCCGAGGACAAGGTG-1’<br>3’-CGTGTACAAGTCTGCGTCCT-2’	Forward<br>Reverse	NM_170735
GDNF	5’-CCAACCCAGAGAATTCCAGA-3’<br>3’-AGCCGCTGCAGTACCTAAAA-4’	Forward<br>Reverse	NM_000514
HLA-G5	5’-AGTCTTCCCTGCCCACCAT-3’<br>3’-CTTTCTCCACAGCACAGCAG-2’	Forward<br>Reverse	NC_00006
IDO	5’-CAAAGGTCATGGAGATGTCC-1’<br>3’-CCACCAATAGAGAGACCAGG-2’	Forward<br>Reverse	NM_002164
IL-10	5’-ACCTGCCTAACATGCTTCGAG-1’<br>3’-CTGGGTCTTGGTTCTCAGCTT-2’	Forward<br>Reverse	NM_000572
MHC II	5’-AGGCAGCATTGAAGTCAGGT-1’<br>3’-GGCAGGTGTAAACCTCTCCA-2’	Forward<br>Reverse	NM_002124
Nanog	5’-CAGAAGGCCTCAGCACCTAC-5’<br>3’-ATTGTTCCAGGTCTGGTTGC-6’	Forward<br>Reverse	NM_024865
NGF	5’-ATACAGGCGGAACCACACTC-3’<br>3’-GCCTGGGGTCCACAGTAAT-5’	Forward<br>Reverse	NM_002506
Oct4	5’-GACAGGGGGAGGGGAGGAGCTA-5’<br>3’-CTTCCCTCCAACCAGTTGCCCCAA-6’	Forward<br>Reverse	NM_002701
Sox2	5’-AATGCCTTCATGGTGTGGTC-5’<br>3’-CGGGGCCGGTATTTATAATC-6’	Forward<br>Reverse	NM_003106
VEGF	p.5’-CTACCTCCACCATGCCAAGT-1’<br>p.3’-ACACAGGACGGCTTGAAGAT-2’	Forward<br>Reverse	NM_001033756
